# The evolutionary origin of digit patterning

**DOI:** 10.1186/s13227-017-0084-8

**Published:** 2017-11-21

**Authors:** Thomas A. Stewart, Ramray Bhat, Stuart A. Newman

**Affiliations:** 1 Department of Ecology and Evolutionary Biology, Yale University, 300 Heffernan Dr, West Haven, CT 06515 USA; 2 Minnesota Center for Philosophy of Science, University of Minnesota, 746 Heller Hall, 271 19th Ave. S, Minneapolis, MN 55455 USA; 3 Department of Molecular Reproduction, Development, and Genetics, Indian Institute of Science, Biological Sciences Building, Bengaluru, 560012 India; 4 Department of Cell Biology and Anatomy, New York Medical College, 40 Sunshine Cottage Rd, Valhalla, NY 10595 USA; 5 Present Address: Department of Organismal Biology and Anatomy, The University of Chicago, 1027 E 57th St, Chicago, IL 60637 USA

**Keywords:** Development, Fin, Genetics, Novelty, Turing, Self-organization

## Abstract

The evolution of tetrapod limbs from paired fins has long been of interest to both evolutionary and developmental biologists. Several recent investigative tracks have converged to restructure hypotheses in this area. First, there is now general agreement that the limb skeleton is patterned by one or more Turing-type reaction–diffusion, or reaction–diffusion–adhesion, mechanism that involves the dynamical breaking of spatial symmetry. Second, experimental studies in finned vertebrates, such as catshark and zebrafish, have disclosed unexpected correspondence between the development of digits and the development of both the endoskeleton and the dermal skeleton of fins. Finally, detailed mathematical models in conjunction with analyses of the evolution of putative Turing system components have permitted formulation of scenarios for the stepwise evolutionary origin of patterning networks in the tetrapod limb. The confluence of experimental and biological physics approaches in conjunction with deepening understanding of the developmental genetics of paired fins and limbs has moved the field closer to understanding the fin-to-limb transition. We indicate challenges posed by still unresolved issues of novelty, homology, and the relation between cell differentiation and pattern formation.

## Background

**Fig. 1:**
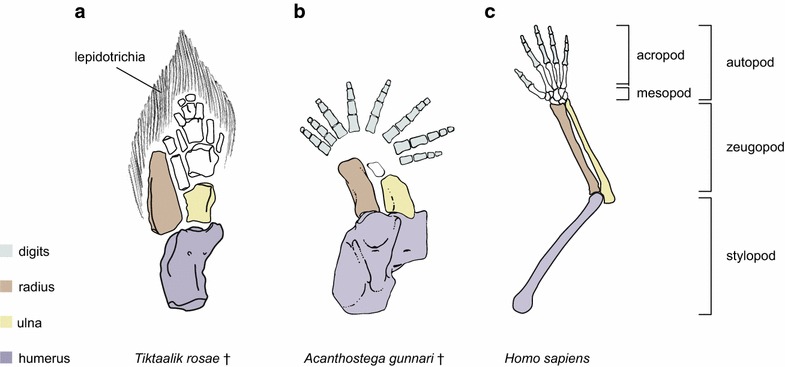
Fin-to-limb transition involved a suite of anatomical changes including loss of dermal fin rays and the acquisition of digits. **a** Pectoral fin skeleton of *Tiktaalik roseae*, an elpistostegid fish. The fin contains both dermal skeleton (lepidotrichia) and endochondral skeleton. Illustration modified from Shubin et al. [64]. **b** Forelimb skeleton of *Acanthostega gunnari,* a stem tetrapod. The limb exhibits a polydactylous pattern, which is characteristic of the earliest limbs. Illustration modified from Coates et al. [65] follows that labeling scheme, although other labeling schemes have been proposed for autopodial elements (e.g., [66]). **c** Forelimb skeleton of human (*Homo sapiens*). This limb shows a pentadactyl pattern and also mesopodial (wrist) elements; these features characterize the crown-group tetrapod condition. Illustration modified from Owen [67]. For each illustration, anterior is oriented to the left. Extinct taxa are noted with a dagger (†)

The appearance of tetrapods in the Late Devonian [1–3] marked a major transition in life’s history, foreshadowing a restructuring of the earth’s ecosystems. Tetrapods are now abundant in terrestrial, aerial, and aquatic environments, and they include both the largest and smallest living adult vertebrates [4, 5]. Likely key to this radiation was the origination and diversification of limbs. The limbs of tetrapods evolved from paired fins, and they can be diagnosed by the absence of dermal skeleton (lepidotrichia) and the presence of digits, which are parallel, non-branching and segmented endoskeletal elements at the distal end of vertebrate paired pectoral and pelvic appendages (Fig. 1) [6]. Over the past few years, hypotheses of how limbs evolved from fins have been restructured dramatically. This progress reflects greater understanding of both the evolution of gene regulation and the role of Turing-type self-organizing systems in the patterning of condensing limb-bud mesenchyme.

Digits develop in a domain of the limb bud marked by late-phase expression of *Hoxa13* and *Hoxd13* [7, 8] and the absence of *Hoxa11*, which is expressed more proximally [9]. Previously, late-phase *Hox* expression was taken as a hallmark of the autopod, and digit origin was therefore attributed to the evolution of a new gene regulatory state in the distal limb-bud mesenchyme [10, 11]. However, reevaluation of actinopterygian [12–14], chondrichthyan [15], and sarcopterygian [16] paired fin development revealed patterns of *Hox* gene expression similar to the late phase of limbs. These patterns are driven in fins and limbs by conserved gene regulatory elements [17, 18]. Most recently, cell lineage tracing and the application of CRISPR/Cas9 editing in zebrafish (*Danio rerio*) showed that *Hox13*-expressing cells of the distal mesenchyme in paired fins form the dermal fin skeleton [19]. Thus, digit origin appears to have involved eliciting new or latent chondrogenic potential of the *Hox13*-expressing cells, transforming the fate and patterning of this compartment of distal fin mesenchyme from fin rays to digits. Loss of the embryonic fin fold in paired appendages might have contributed to this transformation; if mesenchymal cells could no longer migrate into the fin fold, then they would have remained in a terminal position within the fin bud and in a developmental context that promotes differentiation into endoskeleton [12].

The limb skeleton develops by the condensation of mesenchyme in the limb bud [20]. The stylopod (humerus/femur) forms first, followed by the zeugopod (ulna and radius/tibia and fibula), and finally the autopod (the wrist/ankle and digits). Beginning in the 1970s, models for limb development were proposed that predicted that the patterning of the limb skeleton had a causal basis in reaction–diffusion phenomena [21, 22] famously expounded by the mathematician A.M. Turing in a 1952 paper, “The Chemical Basis of Morphogenesis” [23]. In this paper, we review how Turing-type models are being used to explain the fin-to-limb transition. We also indicate future research strategies afforded by these models and explore the conceptual implications of integrating approaches from biological physics and developmental genetics to the questions of limb origination and evolution.

### Turing-type mechanisms of pattern formation

Toward the end of his life, the computer science pioneer A.M. Turing published a paper [23] that addressed an unconventional side-interest of his—biological patterns, phenomena such as the pigment stripes on a zebra’s skin and the seed spirals of the sunflower’s face. The class of mechanisms he advanced was based on simple chemistry and physics, reaction and diffusion, but led to spatial distributions of reagents and products that, counterintuitively, exhibited reproducible spatial non-uniformities. A straightforward way this can occur is if a chemical that activates its own production diffuses from its site of production more slowly than a second chemical, which is also produced in response to the first. If the second molecule inhibits the initial auto-activating reaction, it will act as a “lateral inhibitor,” causing centers of production to form in a spatially separated fashion. Rates of reaction and diffusion define the magnitude of this spacing, which (under an appropriate range of conditions) will be regular: a “chemical wavelength.”

The recognition that the change of cell state (e.g., determination and differentiation), along with secretion of proteins and other molecules, provides a biological analogue of a chemical reaction, while any transport of molecules across a tissue can be treated formally like diffusion, helped connect experimental developmental genetics to abstract mathematics. Moreover, as Meinhardt noted [24], activator–inhibitor networks described above are not the only mode by which reaction–diffusion systems can break spatial uniformity and create patterns. Substrate–depletion networks employ the local breakdown of a precursor of the autocatalytic activator as the causal basis of the spacing of primordia. Other network topologies with indirect activation or inhibition circuitry can similarly produce patterns [25].

One important feature that distinguishes tissue-based developmental systems from the chemical systems that can also sustain reaction–diffusion patterning is the potential ability of “reaction” or signaling centers (i.e., groups of cells) to move relative to one another. Classic Turing-type mechanisms typically induce morphogenetic changes only after a molecular pattern has been set. They have therefore been placed in the category of “morphostatic” developmental mechanisms [26]. However, it is also possible for cell rearrangement to occur simultaneously with the establishment of patterns [27]. Indeed, such rearrangement can even be required for pattern formation [28, 29]. Turing-type models that include such cellular movement are termed “morphodynamic” [26].

The tetrapod limb, particularly the autopod, has quasiperiodic features that lend its development to being conceptualized as a Turing-type patterning process. Since similar repetitive arrangements can also be discerned in the fins of sarcopterygians and more distantly related chondrichthyans and actinopterygians, there is new consideration of scenarios of limb origin and limb and fin diversification that focus on this class of mechanism and the evolution of their postulated molecular components.

### Turing-type models and the fin-to-limb transition

Turing-type models of limb development purport to account for the autopodial ground plan, with its regularly spaced rods and nodules of cartilage, by the self-organizing capacity of prechondrogenic limb mesenchyme [30–33]. Invariably, the proposed components of such skeletal patterning mechanisms are products of genes deeply conserved across vertebrate phylogeny. These models thus lend themselves to hypotheses of evolutionary transformation. Two recently proposed Turing-type mechanisms of digit patterning are based on experimental findings from two tetrapods (a mammal and a bird) and one cartilaginous fish (a shark) making their conclusions, at least for the present, difficult to generalize [34–36].

Studies of mouse (*Mus musculus*) by Sharpe and colleagues [34] revealed that the spatiotemporal expression of the transcription factor *Sox9*, the master regulator of chondrocyte differentiation [37], is dependent in the autopod upon its interactions with two morphogens, Bmp2 and one or more members of the *Wnt* family [34]. The dynamical interactions of these three factors can be represented in the form of a substrate–depletion Turing-type process, termed the BSW (Bmp-Sox9-Wnt) network [34]. Studies of the BSW network in the embryonic pectoral fins of the catshark (*Scyliorhinus canicula*) showed it to be integral to the formation of an array of cartilage nodules that comprise the distal-most components of the endoskeleton, but not the more proximal parallel rods of cartilage [36]. This was consistent with indications from the mouse experiments [34] that this network is involved in patterning only the digits and not the more proximal skeletal elements.

From these studies, the researchers concluded that the BSW network functions broadly across gnathostomes and that modulation of its parameter values can produce morphologically dissimilar structures [36]. Regarding digit origin, it was hypothesized that a distal array of repeated endoskeletal elements produced by the BSW network was present in paired fins at the base of jawed vertebrates and has been conserved in the paired appendages of chondrichthyans and tetrapods. Given that it can be challenging to diagnose a periodic pattern in the distal-most endoskeleton of some stem tetrapods (e.g., Fig. 1a), it is conceivable that the BSW network was not maintained distally in the paired appendages in early sarcopterygians. If this is the case, then the network was redeployed in the distal fin domain at some point along the tetrapod stem. Perhaps, during such a window where it was not operating in the paired appendages, the network operated elsewhere in the body (e.g., median fins). Recruitment of skeletal patterning systems from one fin to another is not unprecedented—it has been observed repeatedly in adipose fins [38, 39]. It will thus be important to test whether the BSW network patterns the endoskeleton of other fins and limbs to clarify its contribution to digit origin.

**Fig. 2:**
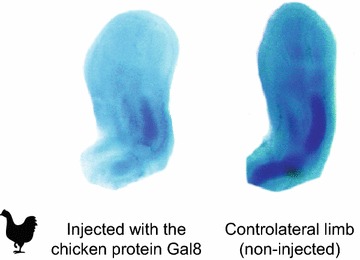
Experimental manipulation of Gal8 affects zeugopod, stylopod, and autopod development in the avian wing. Left—wing bud from a 5-day chicken embryo injected with Gal8 protein, grown for a day and then isolated, fixed, and stained for cartilage with Alcian blue. Right—the control, contralateral wing bud (reflected). Experimental treatment results in complete absence of chondrified primordia in the autopod, dysmorphic, aberrantly arranged primordia in the zeugopod, and a weakly stained, poorly developed stylopod. The control limb shows well-developed and stained cartilage primordia in the stylo-, zeugo-, and autopodial regions. Images adapted from [40]

In another set of studies, in this case on chicken (*Gallus gallus*), Bhat, Newman, and colleagues showed that two members of the galectin family of carbohydrate-binding proteins (Gal1a and Gal8) interact with each other directly, and via cell surface receptors indirectly, to constitute a multiscale “reaction–diffusion–adhesion” Turing-type mechanism for limb skeletal patterning [40]. Gal1a serves as an activator of chondrogenesis and Gal8 an inhibitor, but the ability of the network to form patterns depends on cell movement, making the mechanism morphodynamic [28]. Although evidence for the role of the two-galectin network derives predominantly from in vitro and in vivo manipulations of autopodial mesenchyme, localization and manipulation studies suggest that the mechanism also acts more proximally, patterning the zeugopod and stylopod (Fig. 2).

The two-galectin network has been represented by a mathematical model that predicts the number of well-spaced foci of cartilage that will form according to the values of experimentally determined parameters and variables [28, 40–42]. The limb pattern, with its general increase in the number of parallel rods along the proximodistal axis, is predicted to depend on the capacity of Gal1a and Gal8 to compete for a common cell surface receptor in the limb-bud mesenchyme, and the modulation of the levels of the galectins during development. Regarding the latter, a conserved noncoding motif with binding sites for transcription factors associated with limb development was identified upstream of *Gal8* in sarcopterygians [42], and it could allow for down-regulation of *Gal8* in the apical mesenchyme as the limb bud extends. Assuming the presence of permissive levels of Gal1 protein, this decrease would produce an increasing number of cartilage elements as the limb grows (one stylopod, two zeugopodial elements, and several autopodial elements) [42]. Transcription factors with putative binding sites within the conserved noncoding motif include *Meis1* (necessary for determination of proximal limb elemental identities [43]), *Tcfcpl1* (a transcription factor expressed in murine limb musculogenesis [44]), and Runx1 and Runx2 (required for differentiation of chondroprogenitor cells to chondrocytes and for chondrocytic maturation, respectively [45, 46]).

The evolution of the two-galectin patterning system has been studied by comparative genomic and protein structural analyses. All gnathostomes analyzed except for the African coelacanth (*Latimeria chalumnae*) possess an ortholog of *Gal1* that is putatively chondroinductive [41]. The coelacanth does have the paralogous galectin, *Gal2*, whose product has modest *Gal1*-like activity [40] and which might serve a similar function in that species. Although experimental data are only available from the chicken [40], a duplication of *Gal1* in the sauropsids resulted in a closely related isoform (Gal1b) with substantially less chondroinductive activity [40], and this permits strong inferences on which Gal1s of other species are likely to be chondrogenic [41].

Gal8, which evolved at the base of chordates, is predicted to have a structure that would allow for it to compete for binding with chondrogenic Gal1 protein in all chondrichthyans and sarcopterygians assayed [35]. This competitive potential is not conserved among actinopterygians [35]. This suggests that the potential to produce periodic skeletal elements by this patterning network originated in the gnathostome stem and that it has been lost in some actinopterygians.

Thus, the two-galectin network is hypothesized to pattern paired fin endoskeleton across jawed vertebrates, with paired fin and limb endoskeletal diversity evolving by species navigating the two-galectin “parameter space” [28]. The origin of the limb pattern, with its highly conserved proximodistal increase in parallel elements [20] (a pattern considered remarkable by Darwin [47]), can be explained by refinement of an ancestral patterning network by the quantitative modulation of Gal8 during limb-bud outgrowth (see refs. [29] and [35]). Future work should test these hypotheses by manipulation and localization of two-galectin gene products in other species. The lack of an observed limb phenotype in *Gal1* null mutant mice [48] is a challenge to the model that needs to be addressed. It is plausible that Gal2 (as proposed for coelacanth) or a mammalian galectin not present in birds (e.g., Gal7) might play a compensatory role.

### The evolution of fin and limb disparity

Presently, the generalizability of the BSW and two-galectin models across vertebrate clades is unknown, as is whether the two mechanisms share an evolutionary relationship to one another. However, their connection to specific genes allows for the formulation of testable hypotheses. For example, do the paired fin endoskeletons of teleosts develop with Turing-type patterning? And are these networks tuned locally across the limb to generate disparate, clade-specific morphologies?

In zebrafish, a teleost, the proximal elements of the pectoral fin endoskeleton form by the perforation and subdivision a single embryonic endochondral fin disk [49]. The fin endoskeleton develops from lateral plate mesoderm cells, which converge to form the fin bud, and the ablation of either anterior or posterior cells of this population causes the loss of associated anterior or posterior skeletal elements [50]. This suggests that mesenchymal regionalization begins before self-organization might occur in the fin bud. The BSW network is not predicted to operate in teleost fishes [36]. However, the two-galectin model is proposed to do so [42] and can account for the development of a cartilage plate of the larval fish as the formation of confluent cartilaginous rods, which can be secondarily subdivided by apoptosis.

To discriminate between these hypotheses, significantly more comparative experimental data are needed for actinopterygians. The diversity of ways by which endoskeletal elements can develop reinforces the need for appropriate selection of developmental models. For example, the propterygium of zebrafish develops by secondary subdivision of larger cartilage plate [49], while in paddlefish it develops as a single condensation [51], and in sturgeon it develops by fusion of two condensations [52]. Turing-type models should be studied in non-teleost actinopterygians (e.g., *Polyodon spathula* and *Lepisosteus oculatus*) and also evaluated in median fins.

Limbs have repeatedly evolved to have digits that are highly differentiated morphologically. Paradigmatic cases include the aye–aye (*Daubentonia madagascariensis*) forelimb, which bears a long and gracile D3 for high-frequency tapping, and the pterosaur forelimb, which has a dramatically elongated D4 to support the flight membrane. These examples show how the plesiomorphic autopod, with many high-fidelity serial homologs, has evolved in various lineages to be characterized by digits with disparate phenotypes.

Differences between digits can be apparent when their primordia first condense. Within an autopod, digit condensations can be of non-uniform thickness and spacing, with early differences corresponding to adult limb morphologies (e.g., the chicken forelimb [53]). This suggests that patterning networks are modulated locally across the autopod and contribute to the evolution of digit-specific phenotypes [54, 55]. It has been shown that other expressed genes of the mesenchyme can affect these patterning systems. Using a combination of genetic manipulation of *Hoxa13* and *Hoxd11*–*Hoxd13* genes in mouse embryos and limb tissues, and computational modeling, Sheth and coworkers concluded that the products of these genes regulate digit patterning by controlling the wavelength of a Turing-type mechanism [56], a result predicted earlier on the basis of cell biological evidence [57]. Perhaps the distal mesenchyme of fins is similarly tuned; *Hox13* could also affect patterning dynamics of this tissue and contribute to the thin spacing of dermal skeletal fin rays. Additionally, mathematical analysis by Glimm and colleagues showed how an external gradient (e.g., Shh from the zone of polarizing activity [58]) can change the thickness, spacing, and number of parallel stripes produced by a Turing-type mechanism [59]. Moving forward, it will be useful to test whether convergent phenotypes, (e.g., thick median digits found in limbs adapted for digging, such as those of moles and anteaters) evolved in similar ways, for example by alteration of the geometric properties of the limb bud, or molecular gradients, each of which can affect Turing-type patterning processes.

### Are digits novelties?

The question, “how does morphological novelty originate?” has motivated significant research in the field of evolutionary developmental biology. The answers to this question are variable, with the diverse responses reflecting both multiple conceptions of the novelty concept, and disagreements over which factors are regarded as causally relevant for explaining developmental evolution. Regarding the evolution of morphology, the term novelty is usually reserved for new characters, body parts or body plans not diagnosable in out-groups or hypothesized primitive conditions [60–62].

Digits would seem to qualify as novelties; these structures, as defined in introduction, are not present in the paired fins of fishes, and they originated in the tetrapod radiation. However, antecedents to the developmental processes required for digit development (e.g., specification of an autopodial domain and patterning of the autopodial mesenchyme) are observed in the paired fins of fishes. Practitioners of different research approaches (e.g., developmental genetics or biological physics) might disagree on what explanations of homology, and thus novelty, should be based upon. For example, if a core intracellular gene regulatory network (GRN) for specifying an individualized developmental territory is conserved between digits and distal dermal fin rays of paired fins, then it might be said that digits represent only a new “character state” for an existing “character identity.” This perspective might not regard digits as novelties, per se. Conversely, if patterning dynamics (e.g., Turing-type mechanisms) are considered to have causal explanatory power for hypotheses of homology distinct from GRNs, then assertions of homology between lepidotrichia and digits will be understood differently. For example, if dermal fin rays and digits are not patterned by shared derived Turing-type mechanisms, then they would not be regarded as homologous. In the “physicalist” framework, homology claims would be made with reference both to operation of the Turing-type mechanism as a generic physical process (i.e., one that can organize different materials in similar ways [58]) and also the other developmental processes that characterize its specific, local context.

It seems intuitive that a comprehensive understanding of developmental evolution will bring together multiple research approaches and perspectives. However, it is not always clear how such integration should be achieved [63]. Study of limb development and evolution reveals real differences in conceptual frameworks that exist across the Evo-Devo community. Debates about proper framing are not new, and it is unclear whether consensus will be reached on how best to describe the causes or even arrive at a consensual meaning of novelty. Still, questions like “are digits novelties?” usefully focus attention on a specific biological phenomenon and can help to clarify conceptual issues [57].

## Conclusions

Hypotheses for the evolutionary origin of digits should consider two phenomena: the specification of the autopodial domain and the patterning of mesenchyme in this domain. In this paper, we have reviewed recent advances in understanding how digits are patterned as foci of condensed mesenchyme in the autopod and discussed how these models are being applied to inform the fin-to-limb transition. Significant work remains to be done to test hypotheses beyond a few model systems so as to discriminate between model types and to ensure that these conceptual and experimental tools can be further leveraged to analyze the evolution of morphological disparity in fins and limbs.
